# Bilateral Earlobe Crease as a Marker of Premature Coronary Artery Disease

**DOI:** 10.7759/cureus.2616

**Published:** 2018-05-13

**Authors:** Corina Iorgoveanu, Ahmed Zaghloul, Aakash Desai, Anand M Krishnan, Kathir Balakumaran

**Affiliations:** 1 Internal Medicine, University of Connecticut Health Center, Farmington, USA; 2 Cardiology, University of Connecticut Health Center, Farmington, USA

**Keywords:** coronary, bilateral diagonal ear lobe creases, heart diseases, myocardial infarction

## Abstract

Cardiovascular diseases, including heart disease and stroke, are the world’s largest killers. More than 800,000 people die from cardiovascular disease each year in the United States (US). Heart disease is estimated to cost 200 billion US Dollars (USD) annually. Early identification of an inexpensive marker which allows for early intervention is the need of the hour. We present a case describing one such marker which can be easily appreciated on physical examination. Several studies have shown, not only the association between the presence of the diagonal earlobe crease (DELC) and coronary artery disease (CAD) but also a correlation with the extent and severity of CAD, independent of cardiovascular risk factors.

Our patient who had no known CAD or risk factors presented with an acute coronary syndrome (ACS). On exam, he was noted to have bilateral DELC. Over the course of his workup, he was noted to have severe triple vessel disease and eventually underwent surgical revascularization. We seek to increase awareness of this valuable physical sign which has far-reaching consequences in the prognostication and risk stratification of patients.

## Introduction

Cardiovascular diseases, including heart disease and stroke, are the world’s largest killers. Early efforts to diagnose coronary artery disease (CAD) urged clinicians to seek noninvasive markers. One such marker is the diagonal earlobe crease (also known as Frank's sign). This fold or crease in the skin of the ear-lobe, originally described by Sanders T. Frank in 1973, is associated with CAD. Since then, several studies have shown, not only the association between the presence of the diagonal earlobe crease (DELC) and CAD, but also a correlation with the extent and severity of CAD, independent of cardiovascular risk factors. Many individuals suffer adverse events from atherosclerosis without having the classical risk factors like hypertension, hyperlipidemia, diabetes or obesity. Physical signs like DELC can identify individuals at high risk of atherosclerosis [1].

The modern approach to detecting heart disease has likely forgotten the usefulness of the DELC, which may be used to identify those who are at higher risk of CAD. Patients with DELC may be at higher risk for coronary events, even if currently without diagnostic evidence of CAD. Broader recognition among primary care physicians of this easily detectable sign may facilitate early diagnosis in patients at risk for CAD.

## Case presentation

**Figure 1:**
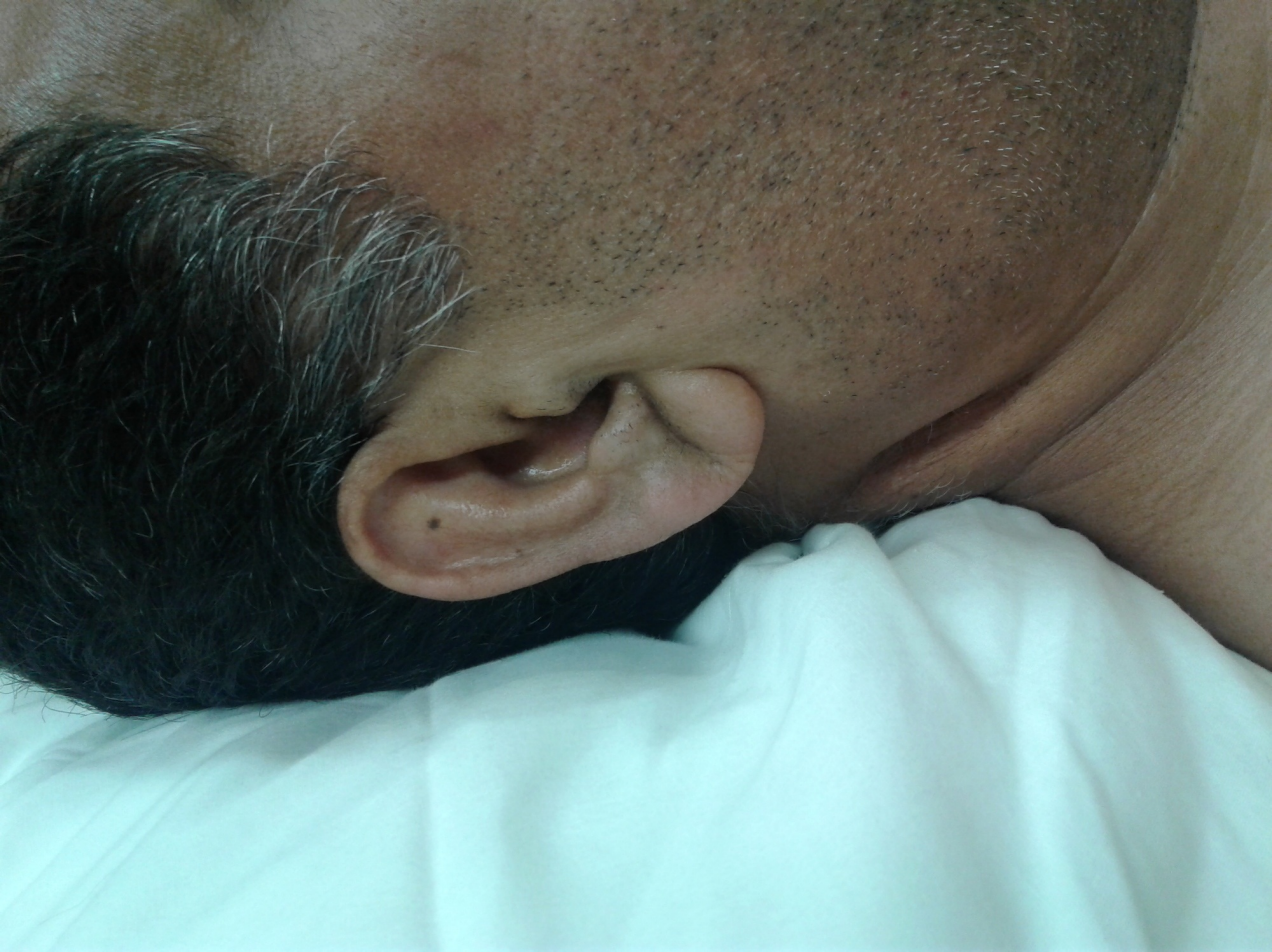
Deep crease running from the lower pole of the external meatus diagonally backwards to the edge of the lobe at approximately 45 degrees, covering at least 2/3rd of its path.

A 44-year-old male with past medical history of hypertension, was in his usual state of health until the morning of admission, when he was awakened by a substernal pressure-like chest pain associated with shortness of breath. He had no prior history of tobacco use, alcohol use, illicit drug use or family history of premature coronary artery disease. On admission, the patient was hemodynamically stable. Physical examination revealed an overweight male with bilateral earlobe creases (Figure 1) and no other relevant findings.

**Figure 2:**
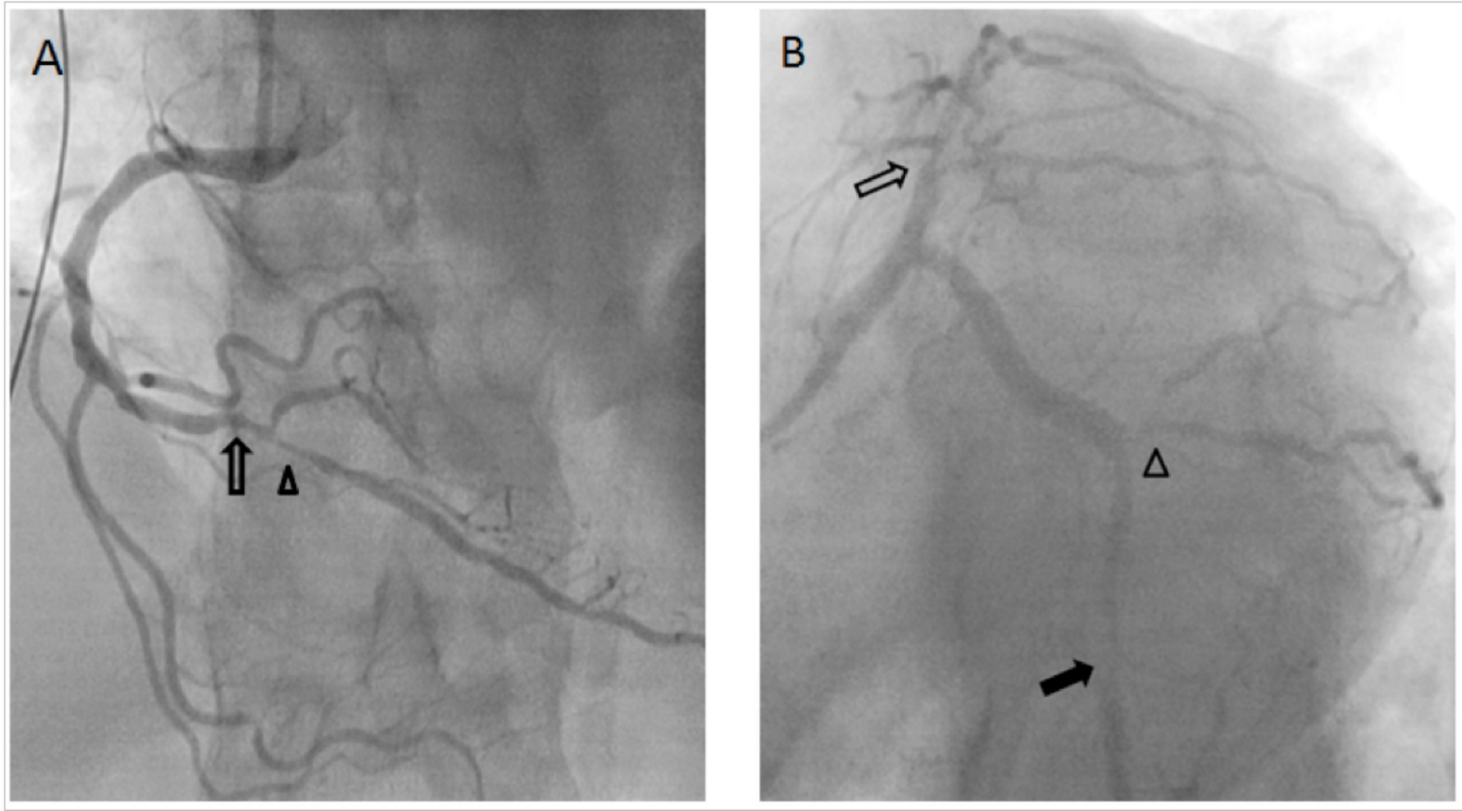
(A) Anterior posterior cranial view of the right coronary artery revealing significant stenosis at the distal right coronary artery (open arrow) and the proximal segment of the posterior descending artery (arrow head). (B) Left Anterior oblique caudal view of the left coronary artery revealing severe stenosis at the mid left anterior descending artery (open arrow), obtuse marginal artery (open arrow head), and distal left circumflex artery (closed arrow).

A 12-lead electrocardiogram (ECG) revealed 2 mm downsloping ST depressions in the inferolateral leads along with 1 mm ST elevation in aVR. Initial cardiac enzymes included a troponin of 0.44 ng/ml, with normal creatine kinase (CK) and creatine kinase-muscle/brain (CK-MB) levels. The patient was given aspirin and intravenous heparin in the emergency room in addition to morphine and sublingual nitroglycerin. He continued to have ongoing pain despite analgesics and nitrates, thus he was transferred to the cardiac catheterization lab where angiography revealed severe triple vessel disease (Figure 2). The decision was made to proceed with coronary artery bypass surgery (CABG). A transthoracic echocardiogram (TEE) revealed hypokinesis of the inferior wall of the left ventricle and ejection fraction of 45%.

## Discussion

The DELC has been described in literature as a surrogate marker that can recognize high-risk patients having occult atherosclerosis. After the initial publication in 1973, many studies have followed and reported the association between DELC and CAD.

The pathophysiological explanation underlying this correlation remains unclear. It has been proposed that the crease is an external sign of a microangiopathic lesion of terminal vessels that occurs in systemic diseases such as CAD. It was originally suggested that both earlobe and heart are supplied by end-arteries, thereby limiting the possibility for collateral circulation [2]. In a Japanese study, Higuchi et al. demonstrated that males with DELC have shortened telomeres. These findings suggested that DELC is a helpful physical exam finding of an accelerated aging process and a marker of high-risk patients. Other studies have suggested that degeneration caused by changes in the collagen to elastin ratio may be the final common pathophysiological pathway of both atherosclerosis and DLEC [3]. These changes were observed in biopsy specimens taken from earlobe creases that depicted vasculature morphology similar to the coronary bed, pathognomonic of CAD [2].

Over the years, several studies have demonstrated that DELCs were independently and significantly associated with an increased prevalence of CAD and carotid arterial thickening. In addition, cohort studies have shown that high-risk participants with unilateral, bilateral, or no earlobe creases, have different prognoses of CAD [1].

A recent study conducted in the US including patients imaged with computed tomography (CT) coronary angiography found that DELC was independently and significantly associated with increased prevalence, extent, and severity of CAD [4].

Another study performed by Hou et al. investigated the role of DELC in the clinical outcome of patients who underwent successful coronary artery drug-eluting stents implantation. In patients with more than four risk factors, the presence of DELC was associated with higher chance of developing major adverse cardiac events after successful percutaneous coronary intervention [5].

Lastly, further research on a larger scale might be indicated to validate the findings described in previous studies, which have generally used smaller sample sizes.

## Conclusions

The DELC has been correlated with significant CAD in the absence of major risk factors. It has been suggested as a simple, noninvasive marker of cardiovascular disease which could alert clinicians to the presence of risk factors. As such, primary practitioners should be aware of this easily detectable sign to facilitate early diagnosis of CAD.
